# Postoperative Recovery Outcomes for Obese Patients Undergoing General Anesthesia: A Meta-Analysis of Randomized Controlled Trials

**DOI:** 10.3389/fsurg.2022.862632

**Published:** 2022-07-28

**Authors:** Zhen-Hua Hu, Zhe Liu, Gai-Fang Zheng, Zhan-Wen Li, Sheng-Qun Liu

**Affiliations:** Department of Anesthesiology, Henan Provincial People’s Hospital, Henan University, China

**Keywords:** obese, general anesthesia, postoperative recovery, meta-analysis, patient

## Abstract

**Purpose:**

This study was performed to assess the postoperative recovery outcomes in obese patients undergoing general anesthesia.

**Methods:**

The eligible studies were identified from PubMed, EmBase, and the Cochrane library until December 2020. The standard mean differences (SMDs) with 95% confidence intervals (CIs) were used to calculate the role of desflurane, sevoflurane, and propofol on recovery outcomes, and the analyses using the random-effects model.

**Results:**

Eleven randomized controlled trials involving 713 obese patients undergoing general anesthesia were selected for final meta-analysis. We noted desflurane was associated with a shorter time to eye-opening than sevoflurane (SMD: −0.86; 95% CI, −1.43 to −0.28; *P* = 0.003). The use of desflurane with shorter time to extubation as compared with propofol (SMD: −1.13; 95% CI, −1.52 to −0.73; *P* < 0.001) or sevoflurane (SMD: −1.19; 95% CI, −2.15 to −0.22; *P* = 0.016), while sevoflurane was associated with longer time to extubation as compared with propofol (SMD: 1.47; 95% CI, 1.03 to 1.91; *P* < 0.001). Desflurane were associated with shorter time to stating name as compared with propofol (SMD: −1.40; 95% CI, −2.32 to −0.48; *P* = 0.003) or sevoflurane (SMD: −2.09; 95% CI, −3.33 to −0.85; *P* = 0.001). In addition, desflurane was associated with a longer time for orientation to place as compared with propofol (SMD: 0.65; 95% CI, 0.22 to 1.07; *P* = 0.003), while desflurane with shorter time for orientation to place as compared with sevoflurane (SMD: −0.88; 95% CI, −1.46 to −0.30; *P* = 0.003).

**Conclusions:**

The use of desflurane could provide better recovery outcomes in obese patients undergoing general anesthesia. Further large-scale trials should be comparison the long-term effectiveness of various anesthetics.

## Introduction

Obesity is considered a major epidemiological problem and the number of obese persons reached over 600 million in 2014 (1). The treatment of obese patients and related complications remains a challenge, especially in anesthesiology. The potential complications of obesity, include insulin-resistance, diabetes, cardiovascular disease, hormonal imbalance, glomerulopathy or neoplasia, and other disorders, which play an important role in respiratory and hemodynamic nature (2–4). The potential adverse effects include obstructive sleep apnoea, hypoventilation syndrome, or postoperative atelectasis (5). Moreover, obese patients were associated with an increased risk of adverse respiratory events after general anesthesia (6). Therefore, obese patients required careful preoperative evaluation and intraoperative management to ensure better recovery outcomes and fewer adverse events (7).

Nowadays, a variety of anesthetics are already used for morbidly obese patients, while no single strategy has shown more beneficial effects than others. Desflurane, sevoflurane, and propofol are widely used for obese patients, and rapid postoperative recovery is related to earlier maintenance of airways and associated with effective protection against aspiration and greater oxygenation (8). However, the use of longer-acting opioids could bias the postoperative recovery outcomes. Numerous studies have already compared the postoperative recovery outcomes of desflurane, sevoflurane, and propofol for obese patients (9–19). We, therefore, performed a systematic review and meta-analysis to compare the effects of desflurane, sevoflurane, and propofol on recovery outcomes for obese patients.

## Methods

### Data Sources, Search Strategy, and Selection Criteria

The reporting and conducting of this study were in accordance with the Preferred Reporting Items for Systematic Reviews and Meta-Analysis Statement (20). The query task for systematic reviews was to collect randomized controlled trials (RCTs) that compared the role of desflurane, sevoflurane, and propofol on recovery outcomes for obese patients undergoing general anesthesia was eligible in this study, and the publication language was not restricted. The potentially relevant articles were searched in PubMed, EmBase, and the Cochrane Library from January 2000 to December 2020, and the following search terms were used through Medical Subject Heading to text words: (“obese” OR “overweight” OR “bariatric surgery” OR “body mass index”) AND (“anesthesia” OR “anesthetic” OR “desflurane” OR “sevoflurane” OR “propofol” OR “total intravenous anesthesia” OR “general anesthesia”). Meta-analyses were screened out if they did not meet the inclusion criteria listed later, first at the title/abstract level, and later at the methods section level if necessary. We also reviewed the reference lists in retrieved studies for any further eligible studies.

The details of inclusion criteria were: (1) Participants: obese patients undergoing general anesthesia; (2) Intervention and control: any 2 of desflurane, sevoflurane, and propofol; (3) Outcome: time to eye opening, time to extubation, time to stating name, time for orientation to place, and time required for hand squeezing; and (4) Study design: the study had to have RCT design. The relevance of studies was assessed by reviewing the title and abstracts, and the full-text evaluations were evaluated to obtain whether potentially relevant trials reported an outcome of interest. The study selection was independently double-checked the inclusion and exclusion criteria by two reviewers, and the conflicts between reviewers were resolved by group discussion until a consensus was reached.

### Data Collection and Quality Assessment

The following data items in each included study were independently extracted by two reviewers: first author’s name, publication year, region, sample size, mean age, male proportion, body mass index (BMI), anesthesia technique, surgical technique, and reported outcomes. Then the quality of each trial was assessed using the Jadad scale by the same two reviewers, which was based on randomization, blinding, allocation concealment, withdrawals and dropouts, and use of intention-to-treat analysis (21). Any disagreement between the two reviewers were settled by an additional reviewer referring to the full text of the articles.

### Statistical Analysis

The effectiveness of a treatment on postoperative recovery outcomes was assigned as a continuous variable, and the standard mean difference (SMD) with a 95% confidence interval (CI) was calculated before data pooling. Then the random-effects model was used to calculate pooled SMD and 95% CI for comparison of the postoperative recovery outcomes after using desflurane, sevoflurane, or propofol (22, 23). Heterogeneity across included trials was evaluated using the *I*^2^ and Q statistic, and the significant heterogeneity was defined as *I*^2^ > 50.0% or *P* < 0.10 (24, 25). Subgroup analyses were also conducted for the postoperative recovery outcomes of desflurane versus sevoflurane according to mean age, male proportion, BMI, and surgical technique, and the difference between subgroup analyses was assessed by using the interaction t-test, which assumed the distribution of effect estimates were normal (26). Publication bias for investigated outcomes was assessed by using the funnel plot, Egger, and Begg tests (27, 28). The P-value for pooled conclusions is 2-sided, and the inspection level was 0.05. All of the analyses in this study were conducted by using the software STATA (version 10.0; Stata Corporation, College Station, TX, USA).

## Results

### Literature Search

**Figure 1:**
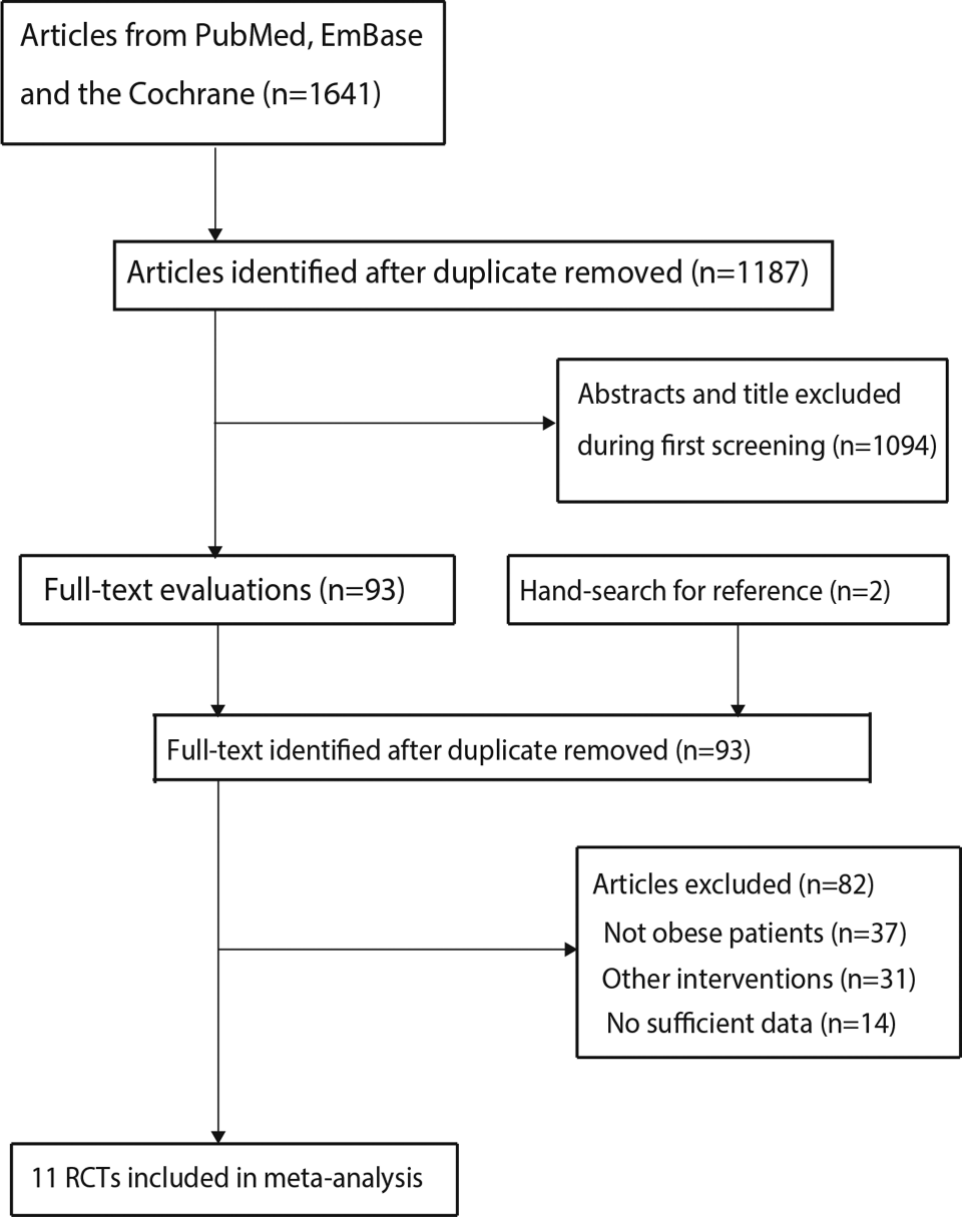
The PRISMA flowchart regarding the trial selection process.

The electronic searches yielded 1,641 articles, and the 1,187 articles were retained after duplicate articles were removed. A total of 1,094 studies were excluded by reviewing their titles and abstracts because they reported irrelevant. The remaining 93 potentially eligible studies were retrieved, and 82 studies were removed after detailed evaluations: patients not obese (*n* = 37), other interventions (*n* = 31), and lacking sufficient data (*n* = 14). Reviewing the reference lists of the remaining studies did not find any new eligible studies. Finally, 11 RCTs were selected for meta-analysis (9–19), and the details of the study selection process are shown in Figure 1.

### Study Characteristics

The characteristics of included studies are summarized in Table 1, and 713 obese patients undergoing general anesthesia were involved. Six studies compared desflurane with sevoflurane, three trials compared desflurane with propofol, and the remaining two trials compared sevoflurane with propofol. The BMI for each trial ranged from 35.3 to 58.0 kg/m^2^, and the sample size ranged from 23 to 183. Eight trials with high quality (three trials had five scores, five trials had four scores), and the remaining three trials with low quality (three trials had three scores).

**Table 1:** The characteristics of identified randomized controlled trials.

Study	Region	Sample size	Mean age (years)	Male proportion (%)	BMI (kg/m^2^)	Anesthesia technique	Surgical technique	Jadad scale
Juvin 2000 (9)	France	23 (12/11)	39.9 (40.1/39.7)	17.4 (25.0/9.1%)	45.4 (46.5/44.3)	Induction with TCI propofol 8 µg/mL and SCC 1.2 mg/kg. Maintained with 50% N2O and D or P by BIS 45–55	Laparoscopic gastroplasty	4
Salihoglu 2001 (10)	Turkey	40 (20/20)	46.5 (47.1/45.39)	52.5 (60.0/45.0)	50.0 (50.0/50.0)	Induction with S breathing, atracurium 0.6 mg/kg, and alfentanil 50 µg/kg. Maintained with 1%–2% S. Induction with P 21 mg/kg perhr, atracurium 0.6 mg/kg, and alfentanil 50 µg/kg. Maintained with P 6 mg/kg perhr	Bariatric operation	3
De Baerdemaeker 2003 (11)	Belgium	50 (25/25)	36.5 (35.0/38.0)	12.0 (8.0/16.0)	41.0 (41.0/41.0)	Induction with TCI remifentanil, propofol 2 mg/kg of IBW, and rocuronium 0.9 mg/kg of IBW. Maintained with D or S by BIS 45–55	Laparoscopic gastroplasty	3
Strum 2004 (12)	USA	50 (25/25)	42.2 (41.4/42.9)	20.0 (24.0/16.0)	53.5 (53.0/54.0)	After epidural catheter placement, induction with fentanyl and propofol, SCC. Maintained with 6% D or 2% S	Open gastrointestinal bypass surgery	4
Arain 2005 (13)	USA	39 (19/20)	61.2 (62.1/60.3)	92.3 (94.7/90.0)	38.1 (38.5/37.7)	Induction with fentanyl at 2 µg/kg, propofol 1.5–2.0 mg/kg, and SCC 1.25–1.5 mg/kg of IBW. Maintained with D or S by BIS 45–50	Elective surgery >2 h	3
La Colla 2007 (14)	Italy	28 (14/14)	37.2 (40.0/34.3)	57.1 (57.1/57.1)	50.6 (53.3/47.9)	TCI remifentanil, then fibreoptic intubation. After intubation, induction with propofol 2 mg/kg. Maintained with 6% D or 2% S by BIS 45–55	Elective biliointestinal bypass surgery	5
Vallejo 2007 (15)	USA	70 (35/35)	43.0 (44.6/41.4)	0.0 (0.0/0.0)	47.5 (47.3/47.6)	Induction with fentanyl 100–250 µg, rocuronium 5 mg, and propofol 2 mg/kg, SCC 15 mg/kg. Maintained with 6% D or 2% S	Laparoscopic gastroplasty	4
Kaur 2013 (16)	India	40 (20/20)	38.6 (37.8/39.5)	32.5 (40.0/25.0)	50.8 (49.2/52.3)	Induction with fentanyl 1–2 µg/kg, propofol 1.0–1.5 mg/kg, and atracurium 0.5 mg/kg. Maintained with N_2_O and D or S by BIS 40–60	Laparoscopic bariatric surgery	4
Siampalioti 2015 (17)	Greece	100 (50/50)	37.8 (39.0/36.5)	31.0 (34.0/28.0)	58.0 (59.0/57.0)	Induction with propofol 2 mg/kg, remifentanil 1 µg/kg and succinylcholine 1 mg/kg and subsequent intubation of the trachea. Maintained with 1%–3% S or P	Bariatric surgery	5
Tabaka 2017 (18)	USA	90 (45/45)	70.2 (69.8/70.6)	44.4 (55.6/33.6)	35.3 (36.5/34.0)	Induction with propofol 1 mg/kg, followed by fentanyl 1–2 mg/kg, and rocuronium 0.4 mg/kg. Maintained with D or P	Total knee replacement	5
Aftab 2019 (19)	Norway	183 (93/90)	44.0 (43.0/46.0)	23.0 (24.0/22.0)	42.0 (43.0/41.0)	Induction with propofol and remifentanil, and remifentanil/desflurane were used. Maintained with D or P	Bariatric surgery	4

### Meta-Analysis

**Figure 2:**
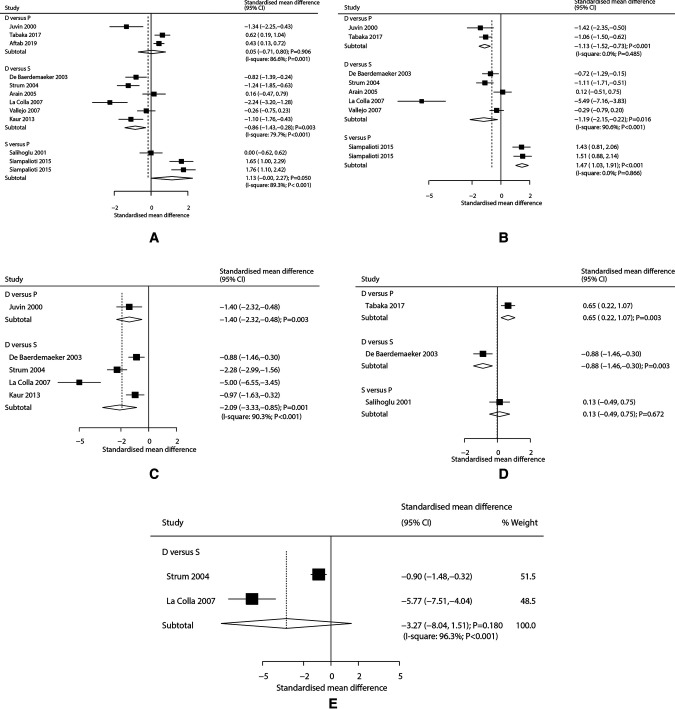
(**A**) Effects of various anesthetics on time to eye opening, (**B**) Effects of varies anesthetics on time to extubation, (**C**) Effects of various anesthetics on time to stating name, (**D**) Effects of various anesthetics on time for orientation to place, (**E**) Effects of varies anesthetics on time required for hand squeezing.

The number of trials for time to eye opening when comparing desflurane with propofol, desflurane with sevoflurane, and sevoflurane with propofol were three, six, and two trials, respectively. We noted desflurane was associated with a shorter time to eye opening as compared with sevoflurane (SMD: −0.86; 95% CI, −1.43 to −0.28; *P* = 0.003; Figure 2A). However, desflurane (SMD: 0.05; 95% CI, −0.71 to 0.80; *P* = 0.906) or sevoflurane (SMD: 1.13; 95% CI, −0.00 to 2.27; *P* = 0.050) were not associated with the time to eye opening when compared with propofol (Figure 2A). There was significant heterogeneity in the comparisons of desflurane with propofol (*I*^2 ^= 86.6%; *P* = 0.001), desflurane with sevoflurane (*I*^2 ^= 79.7%; *P* < 0.001), and sevoflurane with propofol (*I*^2 ^= 89.3%; *P* < 0.001).

The number of trials for time to extubation when comparing desflurane with propofol, desflurane with sevoflurane, and sevoflurane with propofol were two, five, and one trial(s), respectively. We noted desflurane was associated with shorter time to extubation when compared with propofol (SMD: −1.13; 95% CI, −1.52 to −0.73; *P* < 0.001) or sevoflurane (SMD: −1.19; 95% CI, −2.15 to −0.22; *P* = 0.016). However, sevoflurane versus propofol show a longer time to extubation (SMD: 1.47; 95% CI, 1.03 to 1.91; *P* < 0.001) (Figure 2B). There was significant heterogeneity for the comparison of desflurane with sevoflurane (*I*^2 ^= 90.6; *P* < 0.001), while no evidence of heterogeneity for the comparisons of desflurane with propofol (*I*^2 ^= 0.0%; *P* = 0.485), and sevoflurane with propofol (*I*^2 ^= 0.0%; *P* = 0.866).

The number of trials for time to stating name when comparing desflurane with propofol, and desflurane with sevoflurane were one and four trial(s), respectively. We noted desflurane was associated with a shorter time to stating name when compared with propofol (SMD: −1.40; 95% CI, −2.32 to −0.48; *P* = 0.003) or sevoflurane (SMD: −2.09; 95% CI, −3.33 to −0.85; *P* = 0.001) (Figure 2C), and significant heterogeneity for the comparison of desflurane with sevoflurane was observed (*I*^2 ^= 90.3; *P* < 0.001).

Time for orientation to place when comparing desflurane with propofol, desflurane with sevoflurane, and sevoflurane with propofol each had one trial. We noted desflurane was associated with a longer time for orientation to place as compared with propofol (SMD: 0.65; 95% CI, 0.22 to 1.07; *P* = 0.003), while desflurane versus sevoflurane show shorter time for orientation to place (SMD: −0.88; 95% CI, −1.46 to −0.30; *P* = 0.003). However, there was no significant difference between sevoflurane and propofol for the time for orientation to place (SMD: 0.13; 95% CI, −0.49 to 0.75; *P* = 0.672) (Figure 2D). Finally, we noted desflurane was not associated with the time required for hand squeezing as compared with sevoflurane (SMD: −3.27; 95% CI, −8.04 to 1.51; *P* = 0.180; Figure 2E), and significant heterogeneity was observed across included trials (*I*^2 ^= 96.3; *P* < 0.001).

### Subgroup Analysis

Subgroup analyses for the time to eye opening, time to extubation, and time to stating name when comparing desflurane with sevoflurane are shown in Table 2. We noted desflurane versus sevoflurane was associated with a shorter time to eye opening if the mean age of patients <40.0 years, proportion of men <50.0%, BMI ≥ 50.0 kg/m^2^, or patients were treated with gastroplasty. Moreover, desflurane versus sevoflurane was associated with a shorter time to extubation when the proportion of men <50.0%, or patients were treated with gastroplasty. Finally, desflurane versus sevoflurane was associated with a shorter time to stating name in all subgroups.

**Table 2:** Subgroup analyses of desflurane vs sevoflurane for time to eye opening, time to extubation, and time to stating name.

Outcomes	Factors	Groups	SMD and 95% CI	*P*-value	*I*^2^ (%)	*P_Q statistic_*	*P*-value between subgroups
Time to eye opening	Mean age (years)	≥40.0	−0.45 (−1.21 to 0.32)	0.256	81.3	0.005	0.005
		<40.0	−1.30 (−2.03 to −0.56)	0.001	68.0	0.044	
	Male proportion (%)	≥50.0	−1.01 (−3.36 to 1.34)	0.400	94.1	<0.001	0.485
		<50.0	−0.82 (−1.28 to −0.37)	<0.001	59.2	0.061	
	BMI (kg/m^2^)	≥50.0	−1.43 (−2.02 to −0.84)	<0.001	49.7	0.137	<0.001
		<50.0	−0.31 (−0.84 to 0.21)	0.239	61.1	0.076	
	Surgical technique	Gastroplasty	−0.82 (−1.28 to −0.37)	<0.001	59.2	0.061	0.485
		Other Elective	−1.01 (−3.36 to 1.34)	0.400	94.1	<0.001	
Time to extubation	Mean age (years)	≥40.0	−0.43 (−1.10 to 0.24)	0.210	75.6	0.017	0.013
		<40.0	−3.04 (−7.71 to 1.63)	0.202	96.4	<0.001	
	Male proportion (%)	≥50.0	−2.63 (−8.13 to 2.87)	0.348	97.4	<0.001	0.827
		<50.0	−0.68 (−1.15 to −0.21)	0.004	54.0	0.114	
	BMI (kg/m^2^)	≥50.0	−3.23 (−7.52 to 1.07)	0.141	95.8	<0.001	<0.001
		<50.0	−0.31 (−0.76 to 0.14)	0.173	47.1	0.151	
	Surgical technique	Gastroplasty	−0.68 (−1.15 to −0.21)	0.004	54.0	0.114	0.827
		Other Elective	−2.63 (−8.13 to 2.87)	0.348	97.4	<0.001	
Time to stating name	Mean age (years)	≥40.0	−2.28 (−2.99 to −1.56)	<*0.001*	*–*	*–*	0.013
		<40.0	−2.08 (−3.72 to −0.43)	*0*.*013*	*91*.*9*	<*0.001*	
	Male proportion (%)	≥50.0	−5.00 (−6.55 to −3.45)	<*0.001*	*–*	*–*	<0.001
		<50.0	−1.36 (−2.20 to −0.51)	*0*.*002*	*80*.*2*	*0.006*	
	BMI (kg/m^2^)	≥50.0	−2.60 (−4.36 to −0.85)	*0*.*004*	*91*.*7*	<*0.001*	0.008
		<50.0	−0.88 (−1.46 to −0.30)	*0*.*003*	*–*	*–*	
	Surgical technique	Gastroplasty	−1.36 (−2.20 to −0.51)	*0*.*002*	*80*.*2*	*0.006*	<0.001
		Other Elective	−5.00 (−6.55 to −3.45)	<*0.001*	*–*	*–*	

### Publication Bias

The publication bias for time to eye opening, time to extubation, and time to stating name were also evaluated and presented in Supplementary Figures S1–S3. There was no significant publication bias for time to eye opening (*P*-value for Egger: 0.170; *P* value for Begg: 0.373), time to extubation (*P*-value for Egger: 0.417; *P* value for Begg: 0.917), and time to stating name (*P*-value for Egger: 0.073; *P* value for Begg: 0.086).

## Discussion

In this meta-analysis of published RCTs, a total of 713 obese patients undergoing general anesthesia from 11 RCTs were recruited, and the characteristics across included trials were broad. This study found desflurane versus sevoflurane was associated with shorter time to eye opening, time to extubation, time to stating name, and time for orientation to place. Moreover, desflurane versus propofol was associated with a shorter time to extubation or time to stating name, and a longer time for orientation to place. Furthermore, sevoflurane was associated with a longer time to extubation than propofol. Finally, the effectiveness between desflurane and sevoflurane could affect by mean age, male proportion, BMI, and surgical technique.

A prior meta-analysis conducted by Liu et al identified 11 RCTs and found desflurane significantly reduced the time required eye opening, time required for hand squeezing, time required for extubation, and time required for name stating as compared with sevoflurane. Moreover, sevoflurane was associated with a shorter time required for extubation as compared with isoflurane, while no significant difference between sevoflurane and isoflurane for postanesthesia care unit discharge time (29). However, this study did not assess the treatment effectiveness between desflurane and sevoflurane according to patients’ characteristics. The potential heterogeneity across included trials was not explored. We, therefore, conducted a systematic review and meta-analysis to assess the effectiveness of desflurane, sevoflurane, and propofol on postoperative recovery outcomes for obese patients undergoing general anesthesia.

The summary results found desflurane shows better recovery outcomes than sevoflurane, and these results were consistent with prior meta-analyses (30). Although the recovery outcome might be related to the duration of surgery, this impact could be balanced by the duration of surgery being similar between the desflurane and sevoflurane groups. The potential reason for this could be desflurane was associated with a lower solubility in blood, lean tissue, and fat than sevoflurane (31). Moreover, the low blood/gas partition coefficient in desflurane could induce rapid and consistent recovery outcomes in obese patients (32). Subgroup analyses found the beneficial effects of desflurane are more evident in the subgroups of mean age <40.0 years, proportio of men ≥50.0%, BMI ≥ 50.0 kg/m^2^, and patients treated with other elective surgery. The potential reason for this could be the duration of wake in younger patients was more sensitive, and the male patients had stronger restorative ability. Moreover, there was a significant association between sevoflurane use with longer airway reflex recovery time could affect by BMI (33). Finally, the elective surgical are significantly related to the duration of surgery, which might affect the recovery time, and contribute to the significant heterogeneity among included trials.

We noted desflurane was associated with a shorter time to extubation, or time to stating name than propofol, while a longer time for orientation to place for patients who used desflurane was observed. The low solubility of desflurane could explain these results, which suggested less desflurane needs to be released from the tissues and eliminated from the body (34). Moreover, propofol as a lipid-soluble anesthetic and associated with a prolonged effect in obese patients because the proportion of fat in obese patients was high. The longer time for orientation to place could be explained by the fact that only one trial reported this effect, which needed further verification by large-scale RCT (18). In addition, we noted sevoflurane was associated with a longer time to extubation than propofol, which is based on the trial conducted by Siampalioti et al. (17). They point out that the time to eye opening and extubation was shorter when using propofol, while the postoperative recovery was rapid for patients who used sevoflurane.

Several shortcomings of this study should be acknowledged. First, mostly included trials compared the treatment effectiveness of desflurane with sevoflurane, and whether comparing desflurane or sevoflurane with propofol could affect patients’ characteristics needs to be further explored. Second, the heterogeneity across included trials is substantial, and not fully explained by subgroup analyses. Third, patients underwent various surgical techniques, and the duration of surgery was not available in most trials. Fourth is the inherent limitations of meta-analysis based on published articles, including inevitable publication bias, and restricted details analyses.

In conclusion, we noted desflurane was associated with better postoperative recovery outcomes than sevoflurane or propofol for obese patients. Moreover, the effectiveness of sevoflurane versus propofol needs to be further assessed. Furthermore, the treatment effectiveness of desflurane versus sevoflurane on recovery outcome could affect by mean age, proportion of men, BMI, and surgical technique.

## Data Availability

The original contributions presented in the study are included in the article/Supplementary Material, further inquiries can be directed to the corresponding author/s.
