# Supplementary material for: Postoperative Recovery Outcomes for Obese Patients Undergoing General Anesthesia: A Meta-Analysis of Randomized Controlled Trials
Source: Front Surg. 2022 Jul 28;9:862632. doi: 10.3389/fsurg.2022.862632 (PMC9366090; doi:10.3389/fsurg.2022.862632)
Supplement: Supplementary file 1 [file Data_Sheet_1_v1.pdf]

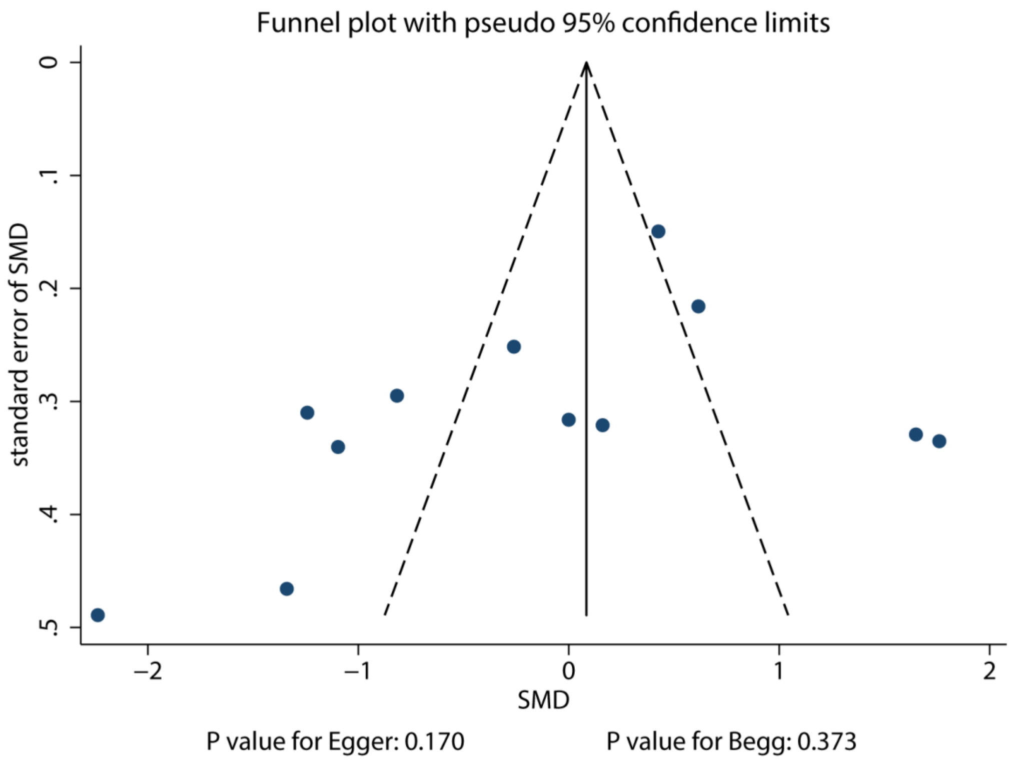

Supplementary Figure 1. Funnel plot for time to eye opening

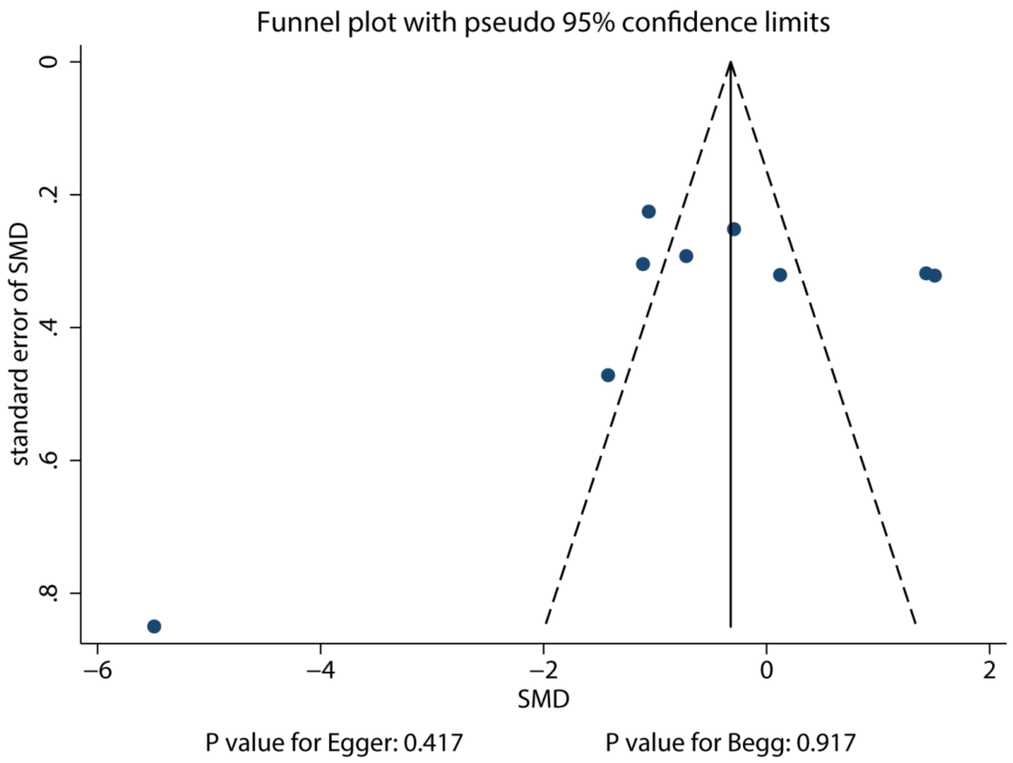

Figure S2. Funnel plot for time to extubation

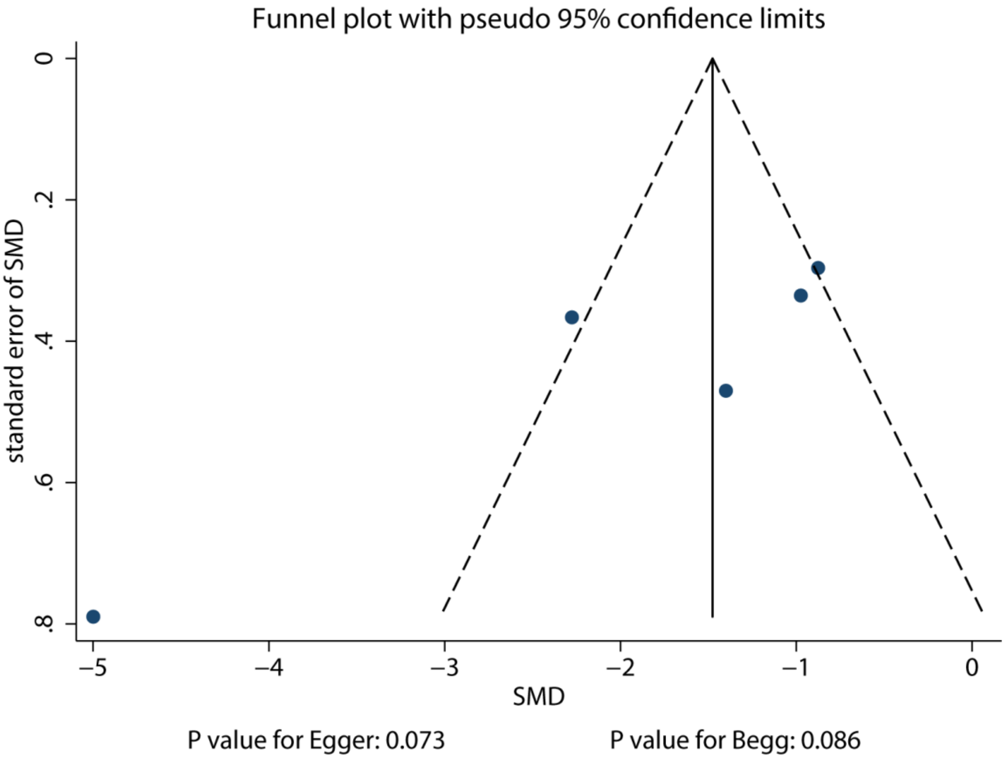

Figure S3. Funnel plot for time to stating name
